# Expression and function of phosphodiesterases (PDEs) in the rat urinary bladder

**DOI:** 10.1186/s12894-017-0244-0

**Published:** 2017-07-07

**Authors:** Xiaofei Zhu, Kui Zhai, Yue Mi, Guangju Ji

**Affiliations:** 1 Department of Urology, Beijing Jishuitan Hospital, Beijing, China; 2 National Laboratory of Biomacromolecules, Institute of Biophysics, Chinese Academy of Sciences, 15 Datun Road, Beijing, 100101 China; 3 Department of Urology, Peking University First Hospital, Beijing, China

**Keywords:** Gene expression, Phasic contraction, Phosphodiesterases, Rat urinary bladder

## Abstract

**Background:**

It has been shown that hosphodiesterases (PDEs) play an important role in mediating the smooth muscle tone of rat urinary bladder. However, the gene expression profiles of them were still unknown.

**Methods:**

Urinary bladder Strips were obtained from both neonatal and adult Sprague-Dawley rats. RT-PCR/western blot and organ bath were used to measure the expression and function of PDEs.

**Results:**

Adult rat urinary bladder expressed various PDE mRNA with the following rank order: PDE5A ≈ PDE9A ≈ PDE10A > PDE2A ≈ PDE4A ≈ PDE4D > PDE4B ≈ PDE3B ≈ PDE8B ≈ PDE7A ≈ PDE7B > PDE1A. PDE1B, PDE1C, PDE3A, PDE4C, PDE8A, and PDE11A were not detected. Of interest, the mRNA and protein of PDE3A were significantly decreased in adult rat urinary bladder compared to neonatal rat urinary bladder. Cilostamide, a specific inhibitor for PDE3, significantly inhibited the amplitude and frequency of carbachol-enhanced phasic contractions of neonatal rat bladder strips by 38.8% and 12.1%, respectively. Compared to the neonatal rat bladder, the effect of cilostamide was significantly blunted in adult rat urinary bladder: the amplitude and frequency of carbachol-enhanced phasic contractions were decreased by 13.4% (*P* < 0.01 vs neonatal rat bladder) and 4.4%, respectively. However, the mRNA and the protein levels of PDE3B were similar between neonatal and adult rat bladder.

**Conclusion:**

We found that several PDE isoforms were expressed in the rat urinary bladder with distinct levels. Moreover, we showed that the function of PDE3 was blunted in adult rat bladder likely due to the decreased expression of PDE3A.

## Background

Through mediating cAMP and/or cGMP levels, phosphodiesterases (PDEs) are involved in several physiological processes of urinary bladder. Artim et al. showed that PDE5 mediated the phasic contractions of neonatal rat bladder through nitric oxide-cGMP- protein kinase G pathway [1]. Our previous study found that PDE3 and PDE4 regulated the phasic contractions of neonatal rat bladder through PKG and PKA pathways, respectively [2]. To date, 11 PDE families have been identified [3]: 3 families (PDEs 4, 7, and 8) selectively hydrolyze cAMP, 3 families selectively hydrolyze cGMP (PDEs 5, 6, and 9), and 5 families (PDEs 1, 2, 3, 10, and 11) hydrolyze both cyclic nucleotides with varying efficacies. As some of these PDE families consist of more than one gene and some genes are alternatively spliced, 21 different isoforms and more than 50 variants have been identified [4].

The urinary bladder is the organ that collects urine excreted from kidneys before disposal by urination. One of the most important features of the urinary detrusor smooth muscle is its ability to generate considerable phasic contractions [1, 2, 5]. These spontaneous phasic contractions change markedly with age [5]: during the first 2–3 weeks of life, they are characterized by high-amplitude low-frequency spontaneous contractions; then, they turn into low-amplitude high-frequency pattern characteristic of normal adult rat. The spontaneous activities in neonatal rat bladder are likely to be myogenic [6] and can also be modulated by the interstitial cell network [7–9].

In this study, we tested the expression profiles of all PDEs families except PDE6 (its expression is restricted to the retina) in adult rat urinary bladder with RT-PCR techniques. Moreover, we evaluated the functional role of PDE3 in mediating the phasic contraction of neonatal and adult rat bladder strips through pharmacological method.

## Methods

### Animals and regents

Neonatal (10-day old) and adult (90-day old) Sprague-Dawley rats were purchased from Vital River Laboratories (Beijing, China). All animal procedures described in this study were performed according to the Guide for the Care and Use of Laboratory Animals published by the US National Institutes of Health (NIH Publication No. 85–23, revised 1996), and with approval from the Institute of Biophysics Committee on Animal Care. The rat was anesthetized by 5% chloral hydrate and the whole bladder was removed, placed into cold Tyrode solution composed of (mM): 137 NaCl, 5.4 KCl, 1.8 CaCl_2_, 1.0 MgCl_2_, 10 glucose, 10 HEPES, (pH 7.4). After the cleaning of the fat tissues, the bladder was cut into two longitudinal pieces by using a fine dissecting scissors along the axis from the neck to the fundus.

Cilostamide (Cil) was from Tocris Bioscience (Bristol, UK). All other reagents were from Sigma Aldrich unless mentioned.

### Polymerase chain reaction (PCR)

Reverse transcriptional-PCR were performed as previously reported [2, 10]. Standard PCR was performed using GoldStar Taq DNA Polymerase (CWBIO, Beijing, China) according to the manufacturer’s instructions. The primers sequences were shown in Table 1. PCR conditions were set up as follows: 94 °C for 2 min, then the following three steps for 34 cycles (94 °C for 30 s, 55 °C for 30 s, 72 °C for 1 min), and a final step of cooling to 4 °C. PCR products were run on a 1.7% agarose gel and visualized under UV light using ethidium bromide staining. qRT-PCR was performed on a Corbett Rotor-Gene 6600 QPCR system machine using TransScript™ Green qRT-PCR SuperMix (TRANSGENE BIOTECH, Beijing, China) according to manufacturer’s instructions. The sequences of gene-specific primers were 5′TCACAGGGCCTTAACTTACAC3′ (forward) and 5′GGAGCAAGAATTGGTTTGTCC3′ (reverse) for PDE3A, and 5′GCAAGAGAGAGGCCCTCAG3′ (forward) and 5′TGTGAGGGAGATGCTCAGTG3′ (reverse) for GAPDH. Amplifications were performed with a 10 min template denaturation step at 95 °C, followed by three steps for 35 cycles (denaturation at 95 °C for 30 s, annealing at 58 °C for 30 s, and extension at 72 °C for 30 s). All samples were amplified in triplicate and the mean was obtained for further calculations. The comparative 2^-∆∆Ct^ method was used to analyze mRNA fold changes between neonatal and adult rat. Then the calculated 2^-∆∆Ct^ was transformed into a percentage using the control as 100% to show the mRNA expression difference.

**Table 1:** The primer sequences of PDE isoforms

Name	Sequences
PDE1A (Forward, F)	5′CCACTTTGTGATCGGAAGTC3′
PDE1A (Reverse, R)	5′TTCTGCTGAATGATGTCCACC3′
PDE1B (F)	5′CAGGGTGACAAGGAGGCAGAG3′
PDE1B (R)	5′GACATCTGGTTGGTGATGCC3′
PDE1C (F)	5′TCTCAAAGGATGACTGGAGG3′
PDE1C (R)	5′GCTTCTCTGTCACCCTGTC3′
PDE2A (F)	5′CCTCCTGTGACCTCTCTGACC3′
PDE2A (R)	5′TGAACTTGTGGGACACCTTGG3′
PDE3A (F)	5′TCACAGGGCCTTAACTTACAC3′
PDE3A (R)	5′GGAGCAAGAATTGGTTTGTCC3′
PDE3B (F)	5′CCTCAGGCAGTTTTATACAATG3′
PDE3B (R)	5′TGCTTCTTCATCTCCCTGCTC3′
PDE4A (F)	5′GTGGAGAAGTCTCAGGTGGG3′
PDE4A (R)	5′TGGAACTTGTCAGGCAGGG3′
PDE4B (F)	5′TAGAAGATAACAGGAACTGG3′
PDE4B (R)	5′GCAATGTCTATGTCAGTCTC3′
PDE4C (F)	5′ACGTGGCGTACCACAACAGC3′
PDE4C (R)	5′TACCGCGAGGTGATGGTTCTC3′
PDE4D (F)	5′GGATAATGGAGGAGTTCTTCC3′
PDE4D (R)	5′CGATTGTCCTCCAAAGTGTCC3′
PDE5A (F)	5′CCCTGGCCTATTCAACAACGG3′
PDE5A (R)	5′GTGGGTCAGGGCCTCATACAG3′
PDE7A (F)	5′TGGACAAGCCAAGTGTATGCTG3′
PDE7A (R)	5′TTTAAGTAACAGTGCATGGCC3′
PDE7B (F)	5′AAAGCCCAGTGGAAGAGC3′
PDE7B (R)	5′CGAAGGGAGGTGGTAAATG3′
PDE8A (F)	5′CAACAAGCCTCTGAAAGC3′
PDE8A (R)	5′TCGGTCTGGGAGAAATAC3′
PDE8B (F)	5′ACCACAACTCCACCCATG3′
PDE8B (R)	5′AGAGGCTTGTTGATGCTG3′
PDE9A (F)	5′TGGGTGGACTGTTTACTGG3′
PDE9A (R)	5′CGGTCTTCATTGTCTTTCG3′
PDE10A (F)	5′TGCTTGGTGGCGTTTGTTAG3′
PDE10A (R)	5′TTCTCTGATGCCTGGGATGTAC3′
PDE11A (F)	5′TTTAGCGGTGATTGTGGG3′
PDE11A (R)	5′TCTCGAAGTACAGCGTGAGG3′
GAPDH (F)	5′CAAGTTCAACGGCACAGTCAAG3′
GAPDH (R)	5′GCACCAGTGGATGCAGGGAT3′

### Western blots

Western blots were performed as previously described [2]. In brief, 10 μg protein of each sample was separated by SDS-polyacrylamide gel electrophoresis using 12% gels. The resolved proteins were transferred to PVDF membranes (Millipore) at 300 mA for 45 min or 1 h. The membranes were blocked for 1 h with Tris-buffered saline-Tween 20 (TBST) containing 5% BSA at room temperature with slow shaking. The blocked membranes were probed with polyclonal anti-PDE3A antibody (Santa Cruz Biotechnology, California, USA, 1:1000), polyclonal anti-PDE3B antibody (Santa Cruz Biotechnology, California, USA, 1:3000), and monoclonal anti-GAPDH antibody (Sigma-Aldrich, St. Louis, MO, 1:10,000) at 4 °C overnight. After washing, the membrane was incubated with peroxidase-conjugated IgG (ZSJQ-BIO, Beijing, China, 1:10,000) for 1 h at room temperature. Final detection was performed using enhanced chemiluminescence detection solution 1 and 2 (1:1) (ECL, Millipore, Billerica, MA). Densitometry was used to measure the expression of PDE3A and PDE3B relative to GAPDH or α-actin with ImageJ (NIH).

### Contractile measurement of rat bladder strips

Contractile measurement of rat bladder strips was performed as previously reported [2]. The bladder strips (with intact mucosa) were tied up and mounted in a classical isolated organ bath (coupled the model BL-420F acquisition system, Chengdu TME Technology Co, Ltd., Sichuan, China) to measure isometric tension. The bladder strips were allowed to equilibrate in physiological salt solution [in mM: NaCl 119, KCl 4.7, CaCl_2_ 2.5, MgSO_4_ 1.17, KH_2_PO_4_ 1.18, glucose 11, NaHCO_3_ 25] for 1.5 h before drug testing. After equilibration, 100 nM carbachol was applied to enhance the amplitude and frequency of phasic contractions, without significantly changing the baseline tension, as previously described [1]. The effects of the PDE3 inhibitors, Cil (1 μM), were studied on the carbachol-enhanced phasic contraction. In experiments performed in the presence of pharmacological reagents diluted in dimethylsulfoxide (DMSO), control bladder strips were treated in the presence of the equivalent concentration of DMSO (<0.1%). The mean amplitude and frequency of carbachol-enhanced phasic contractions were during 5 min before or 5 min after application of Cil or DMSO (<0.1%). The effect of Cil on both parameters was expressed in percentage of the amplitude and frequency of carbachol-enhanced phasic contractions measured before drug application. Baseline tone was not appreciably changed by various treatments and therefore was not subjected to detailed analysis.

### Data analysis

Data were represented as mean ± SEM of *n* strips in organ bath experiments or *N* rats in RT-PCR/western blotting experiments. Significant differences were determined by Student’s t-test. Only results with values of *P* < 0.05 were considered significant.

## Results

**Fig. 1:**
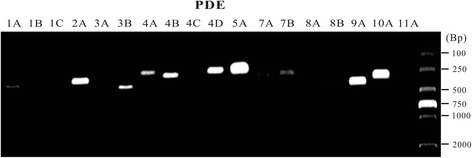
Expression of the mRNA for different PDE isoforms in adult rat urinary bladder. RT-PCR was performed on mRNA extracted from the whole bladder tissues in the presence of primers for PDE1A, PDE1B, PDE1C, PDE2A, PDE3A, PDE3B, PDE4A, PDE4B, PDE4C, PDE4D, PDE5A, PDE7A, PDE7B, PDE8A, PDE8B, PDE9A, PDE10A, PDE11A, and GAPDH. PCR products were resolved by electrophoresis on a 1.7% agarose gel. The representative gels stained with ethidium bromide was shown. *Position* of molecular weight markers is indicated in base pairs (Bp)

Using RT-PCR technique, we determined the mRNA levels of 18 different PDE isoforms in adult rat urinary bladder. As shown in Fig. 1 and Table 2, PDE5A, PDE9A and PDE10A were most highly expressed; PDE2A, PDE4A, PDE4B, PDE4D, and PDE8B were moderately detected; PDE3B, PDE7A, and PDE7B were slightly found; while PDE1B, PDE1C, PDE3A, PDE4C, PDE8A, and PDE11A were absent in all samples tested. Taken together, the mRNA of several PDE isoforms were expressed in rat urinary bladder with distinct levels.

**Table 2:** The expression levels of PDE isoforms in adult rat urinary bladder

	PDE																	
	1A	1B	1C	2A	3A	3B	4A	4B	4C	4D	5A	7A	7B	8A	8B	9A	10A	11A
1	+	˗	˗	++	˗	+	++	++	˗	++	+++	+	+	˗	+	+++	+++	˗
2	+	˗	˗	++	-	+	++	++	˗	++	+++	+	+	˗	+	+++	+++	˗
3	+	˗	˗	++	-	+	++	++	˗	++	+++	+	+	˗	+	+++	+++	˗

+++ indicates high expression; ++ indicates medium expression; + indicates low expression; − indicates no detection

**Fig. 2:**
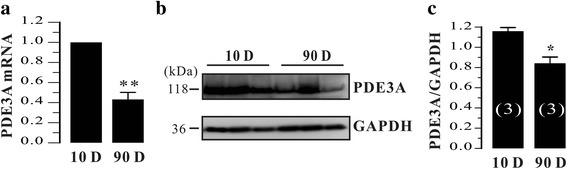
PDE3A expression was determined by qRT-PCR and western blotting in neonatal and adult rat urinary bladder, respectively. **a** Summary data of qRT-PCR from neonatal (*N* = 3) and adult (*N* = 3) rat bladder. **b**-**c** The representative blots and the summary data of these blots are shown. Position of molecular weight markers is indicated in kDa. Data are Mean ± SEM of *N* independent rat bladders. *P < 0.05 and **P < 0.01 are neonatal versus adult

In contrast to our previous finding that PDE3A was highly expressed in neonatal rat bladder [2], PDE3A mRNA was not detected in adult rat urinary bladder. To further verify this, we took qRT-PCR on mRNA from both neonatal and adult rat urinary bladder. As shown in Fig. 2a, the mRNA levels of PDE3A was about 60% decreased in adult rat urinary bladder compared to neonatal rat urinary bladder (*p* < 0.01). Next, we measured its protein levels by using a specific PDE3A antibody. We found that the PDE3A protein was also significantly lowered in adult rat urinary bladder than in neonatal rat urinary bladder (Fig. 2b-c). Our results indicated that PDE3A expression was significantly lower in adult rat urinary bladder than in neonatal rat urinary bladder.

Next, we test the functional roles of PDE3 in the phasic contractions of adult rat urinary bladder smooth muscle. We found that Cil (1 μM), a specific PDE3 inhibitor, significantly decreased the amplitude of carbachol-enhanced contractions of adult rat bladder strips by 13.4 ± 4.0% (*P* < 0.05), and slightly, although not significantly, reduced their frequency by 4.4 ± 4.9%. When comparing to the neonatal bladder smooth muscle strips, Cil-mediated effects on the phasic contractions of smooth muscle strips were much smaller in adult rat urinary bladder. In detail, the effects of Cil were decreased by 2.5 fold (from 38.8% to 13.4%, *p* < 0.01) in amplitude without a significant decrease (12.1% to 4.4%, *p* > 0.05) in frequency. These results suggested that PDE3-mediated functional role was blunted in adult rat urinary bladder.

**Fig. 3:**
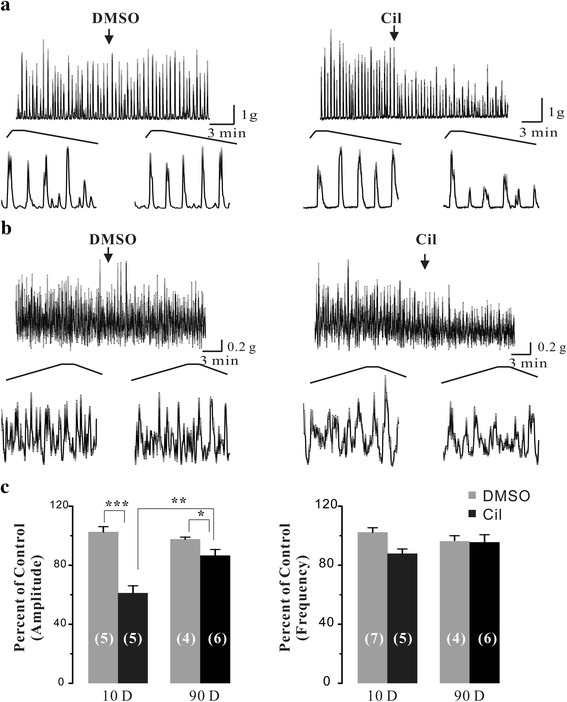
PDE3B expression was determined by RT-PCR and western blotting in neonatal and adult rat urinary bladder, respectively. **a** The representative gels and summary data of RT-PCR were shown. **b** The representative blots and summary data of western blotting were shown. Position of molecular weight markers is indicated in Bp or kDa, respectively. Data are Mean ± SEM of *N* independent rat bladders as indicated in *brackets*

There are two subfamilies of PDE3, PDE3A and PDE3B [11]. They are encoded by different genes and participate in different cellular processes. Is there any relationship between the PDE3B expression and the blunted effects of Cil in adult rat bladder? To answer this question, we tested PDE3B mRNA and protein levels in neonatal and adult rat urinary bladder. As shown in Fig. 3a, PDE3B mRNA was detected in both neonatal (*N* = 4) and adult (*N* = 3) rat urinary bladders. However, the expression level was not significantly different between those two groups (Fig. 3a). Moreover, PDE3B protein expression in neonatal and adult urinary bladders was not different (*N* = 3 each; Fig. 3b). Taken together, PDE3B was not altered between neonatal and adult rat urinary bladder, suggesting that it is not involved in the blunted effects of Cil in adult rat bladder.

## Discussion

Cyclic nucleotide PDEs carry out essential roles in signal transduction by modulating cAMP and cGMP levels and have been recognized as potential targets of several bladder diseases such as overactive bladder. The presence and activities of PDE1–5 have been tested and confirmed in the rodent, rabbit, guinea pig, and human urinary bladder by pharmacological, biochemical, immunohistochemical and molecular methods [12–16]. By using qPCR technique, Lakics et al. have shown that several PDE isoform of PDE7–10 were expressed in the human urinary bladder [16]. Moreover, the expression of PDE5 in different cell types of urinary bladder such as the urothelium, suburothelial interstitial cells and bladder blood smooth muscle cells has been determined in the guinea pig [17]. However, the molecular levels of the whole PDEs family in the rodent urinary bladder are still unknown. Here, we established the mRNA expression levels of 18 different PDE isoforms in rat urinary bladder by RT-PCR. Our results indicated that PDE5 is one of the major isoform expressed in rat urinary bladder, corresponding with its important role in the urinary bladder [15, 17]. In consistent with the finding in human urinary bladder [14], PDE9A gene was shown to be highly expressed in rat urinary bladder. Meanwhile, Nagasaki et al. showed that PDE9 protein was widely distributed in the urothelial epithelium of the human lower urinary tract including the bladder [18]. Thus, it would be of interest to test its functional roles in the bladder. Although PDE10A gene was abundantly expressed, little is known about its role in the rat and human urinary bladder. It has been well known that PDE1 was a major isoform expressed the in human urinary bladder [14]. However, the function of PDE1 in the rat urinary bladder was still controversial. Qiu et al. showed that PDE1 inhibitor had a strong relaxant effect on pre-contracted bladder strips [12]. Our previous study found that PDE1 inhibitor had no effect on spontaneous phasic contraction of bladder strips [2]. One possibility is that the PDE1 inhibitor vinpocetine used in Qiu’s study is not specific to PDE1. It can inhibit voltage-sensitive sodium channels and cause a dose-dependent decrease in extracellular calcium [19]. Moreover, our results indicated that only PDE1A was slightly expressed while both PDE1B and PDE1C were not detected, suggesting that PDE1 expression is varied among species. This difference might partially explain that PDE1 inhibitors exhibit effects in human [20–22] but not in rat bladder [2].

The PDE3 family contains two subfamilies, PDE3A and PDE3B, which are encoded by distinct but related genes [11]. PDE3A and PDE3B isoforms are widely expressed but also exhibit tissue-specific patterns. For example, PDE3A is highly expressed in cardiovascular system, platelets, and oocytes, while PDE3B is mainly expressed in adipocytes, hepatocytes, and pancreatic β-cells [11]. In the present study, we found that both mRNA and protein of PDE3A were dramatically decreased in adult rat urinary bladder (Fig. 2), whereas PDE3B was not altered (Fig. 3). Although the physiological significance underlying in this process are unknown, we speculated that PDE3A might play an important role in the development of rat urinary bladder. It has been established that PDE3A is involved in smooth muscle phenotype switch [23–25] and regulates VSMC cycle progression through controlling cAMP pools [26]. In addition, neonatal urinary bladder is much more proliferative than adult urinary bladder [27]. Thus, it is not surprised that PDE3A would be an important regulator in the development of urinary bladder. However, this possibility should be further tested.

**Fig. 4:**
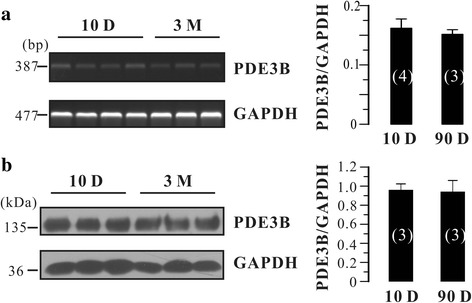
Effects of Cil on the carbachol-enhanced phasic contractions of neonatal and adult rat urinary bladder strips. **a** Original traces of carbachol-enhanced phasic contractions in neonatal rat bladder strips before and after the application of DMSO (<0.1%) and the PDE3 inhibitor Cil (1 μM). **b** Original traces of carbachol-enhanced phasic contractions in adult rat bladder strips before and after the application of DMSO (<0.1%) and the PDE3 inhibitor Cil (1 μM).The *arrows* indicate the times of drugs application. **c** Summary data showing the average effect of DMSO (vehicle) and Cil on the carbachol-enhanced phasic contractions amplitude and frequency. Data are expressed as mean ± SEM of n independent bladder strips as indicated in *brackets*. *P < 0.05 **P < 0.01 and ***P < 0.001 as indicated

There is evidence that the characteristics of bladder spontaneous activity are age-dependent [28], and are related to changes in expression of several proteins such as large conductance Ca^2+^-activated K^+^ channels [5] and connexin [29]. Our results showed that Cil-mediated effects on the phasic contractions were blunted in adult rat bladder strips (Fig. 4) likely due to decreased PDE3A expression. Although PDE3B was consistently expressed throughout the development (Fig. 3), it could not compensate for the PDE3A-mediated effects. These results suggest that PDE3A and PDE3B may take different roles in mediating urinary bladder smooth muscle tone. Nevertheless, these results supported that the notion that PDE3A and PDE3B exhibit distinct functional roles.

## Conclusion

In conclusion, our results for the first time provided a comprehensive view of the gene expression profiles of PDE isoforms in rat urinary bladder. We also found that the function of PDE3 was blunted in adult rat bladder likely due to the decreased expression of PDE3A but not PDE3B, suggesting that PDE3A and PDE3B might play different roles in the urinary bladder.

## Abbreviations

Cil: Cilostamide
DMSO: Dimethylsulfoxide
PCR: Polymerase chain reaction
PDE: Phosphodiesterase
