# Erratum to: Expression and function of phosphodiesterases (PDEs) in the rat urinary bladder

**DOI:** 10.1186/s12894-017-0259-6

**Published:** 2017-08-25

**Authors:** Xiaofei Zhu, Kui Zhai, Yue Mi, Guangju Ji

**Affiliations:** 1 Department of Urology, Beijing Jishuitan Hospital, Beijing, China; 2 National Laboratory of Biomacromolecules, Institute of Biophysics, Chinese Academy of Sciences, 15 Datun Road, Beijing, 100101 China; 3 Department of Urology, Peking University First Hospital, Beijing, China

## Erratum

After publication of this work [1] it was noticed that the figure legends for Figs. 3 and 4 were accidentally swapped.

The original article was corrected.

The publisher apologises for this error.
